# Synergistic killing effects of PD-L1-CAR T cells and colorectal cancer stem cell-dendritic cell vaccine-sensitized T cells in ALDH1-positive colorectal cancer stem cells

**DOI:** 10.7150/jca.62123

**Published:** 2021-09-13

**Authors:** Liu Liu, Yuanyuan Liu, Yang Xia, Guanlong Wang, Xiushan Zhang, Huan Zhang, Yang Xu, Yuan Yuan, Shangquan Liu, Yi Wang

**Affiliations:** 1 The First People's Hospital of Hefei/The Third Affiliated Hospital of Anhui Medical University, Hefei 230061, P.R. China.; 2 Taizhou People's Hospital/The Fifth Affiliated Hospital of Nantong University, Taizhou 225300, P.R. China.; 3 Linquan County People's Hospital, Linquan 236400, P.R. China.

**Keywords:** colorectal cancer stem cells, PD-L1-CAR T cells, colorectal cancer stem cell-dendritic cell vaccine, synergistic killing, ALDH1

## Abstract

Cancer stem cells (CSCs) are characterized by self-renewal and unlimited proliferation, providing a basis for tumor occurrence, metastasis, and recurrence. Because CSCs are highly resistant to conventional chemotherapy and radiotherapy, various immunotherapies, particularly chimeric antigen receptor T cell (CAR-T) therapy and dendritic cell (DC)-based vaccine therapy, are currently being developed. Accordingly, in this study, we evaluated programmed cell death ligand-1 (PD-L1) expression in colorectal CSCs (CCSCs) and non-CCSCs and designed a combination immunotherapy synchronously utilizing PD-L1-CAR-T cells together with CCSC-DC vaccine-sensitized T cells for the treatment of colorectal cancer. PD-L1-CAR-T cells specifically recognized the PD-L1 molecule on CCSCs by binding to the extracellular domain of programmed cell death-1. The CCSC-DC vaccine was prepared using CCSC lysates. We found that aldehyde dehydrogenase 1 (ALDH1)-positive CCSCs were abundant in samples from patient tumor tissues and cancer cell lines. Moreover, PD-L1 was highly expressed in ALDH1-positive CCSCs compared with that in non-CCSCs. Monotherapy with PD-L1-CAR-T cells or CCSC-DC vaccine only elicited moderate tumor remission both *in vitro* and *in vivo*. However, combination therapy markedly killed cancer cells and relieved the tumor burden in mice. Our findings may provide a novel strategy for the clinical treatment of colorectal malignancy.

## Introduction

Despite many recent advances in medical technology, colorectal cancer (CRC) remains one of the most common causes of fatalities in both men and women worldwide 1. Cancer stem cells (CSCs) are a small cell subpopulation characterized by self-renewal, infinite proliferation, and multipotent differentiation, thereby exhibiting the capacity to “reform” the original tumor 2. Biomarkers on the surface of CSCs distinguish them from ordinary cancer cells. In colorectal CSCs (CCSCs), the most prominent biomarkers include the cluster of differentiation (CD) 44, leucine-rich repeat-containing G-protein coupled receptor 5, epithelial cell adhesion molecule, CD133, CD166, and aldehyde dehydrogenase 1 (ALDH1) 3. Studies have shown that the majority of cells in tumor masses lack the capacity for self-renewal and are nontumorigenic, whereas CSCs are proposed to be tumorigenic 4, 5.

Conventional chemotherapy and radiotherapy do not often result in complete tumor remission and can cause adverse reactions 6. Currently, several approaches are being explored to develop novel therapies specific to CCSCs. For example, targeting the programmed cell death-1 (PD-1)/programmed cell death ligand-1 (PD-L1) signaling pathway has become a major focus in cancer immunotherapy 7. PD-L1 is critical for tumor cells that evade immune surveillance of organisms by inhibiting T-cell functions via binding to PD-1 on the T-cell surface. Generally, PD-L1 and PD-1 negatively regulate T-cell activation and proliferation, modulating the homeostasis of the immune system 8. However, in tumors, upregulated PD-L1 interacts with PD-1 and forms a complex to deliver an inhibitory signal that negatively regulates the cytotoxicity of T cells and results in T-cell disorder and exhaustion 9. In addition to the known effects of PD-L1 on immune responses, PD-L1 also acts as a “molecular barrier” to protect cancer cells from cytolysis 10.

Adoptive transfer of chimeric antigen receptor T cells (CAR-T cells) has recently emerged as a promising anticancer approach. The CAR-T technique arms T cells with a chimeric antigen receptor, which containing an extracellular antigen recognition domain, a transmembrane domain, and an intracellular costimulatory domain. CAR-T cells can recognize and kill tumor cells that express specific antigens without the restriction of the major histocompatibility complex 11. Currently, CAR-T cell immunotherapy has been shown to have applications in the treatment of hematologic tumors, particularly B lymphocytic leukemia 12. However, the therapeutic effects of CAR-T cells on solid tumors are not ideal owing to the insufficient T-cell migration into tumors and the immunosuppressive microenvironment 13. Nevertheless, CAR-T therapy is still expected to have important applications in cancer immunotherapy.

Designed to evoke tumor-specific CD8^+^ cytotoxic T lymphocyte immune responses, CSC vaccines exhibit excellent performance in clinical practice and have minimal adverse effects 14, 15. Vaccines disrupt the immunological balance between the immune system and the tumor microenvironment, ultimately leading to the elimination of CSCs. However, initial attempts at developing CSC vaccines were unsatisfactory. For example, researchers first inactivated CSCs and injected them into a CRC mouse model, but did not observe satisfactory efficacy 16. Subsequently, researchers combined CSC vaccines with targeted therapy. Indeed, specifically targeting p53, which plays a vital role in regulating the carcinogenicity and metastasis of tumors, reduces the immunotolerance of CSCs and enhances the efficacy of CSC vaccines by stimulating the immune system 17. Moreover, dendritic cells (DCs), which are potent antigen-presenting cells with key roles in both innate and adaptive immune responses 18, have been mixed with inactivated CSCs to produce CSC-DC vaccines; these vaccines were shown to significantly inhibit tumor growth, reduce pulmonary metastasis, and prolong survival, suggesting potential applications in the treatment of CRC 19. However, the roles of these CSC-DC vaccines in CRC have not yet been fully elucidated.

Accordingly, in this study, we aimed to evaluate the roles of PD-L1 in CCSCs. We then designed a combined therapeutic strategy that synchronously transferred PD-L1-CAR-T cells plus CCSC-DC vaccine-sensitized T cells into mice to evaluate its anti-CRC efficacy.

## Materials and Methods

### Patient specimens

We adopted review research method, forty cases of CRC tissues and their paired normal tissues (from January 2017 to December 2018) were used to carry on the analysis. The inclusion criteria were as follows: none of the CRC patients received any preoperative chemotherapy, radiotherapy or immunotherapy; The exclusion criteria were as follows: patients with autoimmune diseases or other types of tumours. All the tissue samples were obtained with the written consent of the patient's family members and the approval of the medical ethics committee. The edges of the tumor tissues were selected for avoiding central necrotic tissues. Para-carcinoma tissues were acquired from normal colorectal mucosa at least a 5 cm distance from the tumor margin. Tumor tissues were used to prepare pathological specimens or single cell suspensions.

### Mice

Thirty-five STOCK-Foxn1 nu/Nju mice (4-5 weeks old) were purchased from Anhui Provincial Hospital. All animals were maintained in a specific pathogen-free environment at the Laboratory Animal Centre of No. 1 People's Hospital of Hefei and used for experiments at 5 weeks of age or older. Mice were monitored frequently to track growth and tumor progression and sacrificed when they became moribund or showed evident weight loss. All animal studies were approved by the Laboratory Animal Centre of No. 1 People's Hospital of Hefei and Use Committee, and the methods were carried out in accordance with approved guidelines. All the mice were anesthetized with 3% isoflurane gas inhalation and sacrificed by cervical dislocation, then tumors were surgically dissected and measured.

### Cell culture

Human CRC cell lines SW480 and HCT116 were purchased from American Type Culture Collection (Manassas, VA, USA). SW480 cells were cultured in Leibovitz's L-15 Medium (Gibco, Life Technologies, Carlsbad, CA, USA) supplemented with 10% fetal bovine serum (Gibco), 100 U/mL penicillin, and 100 μg/mL streptomycin. HCT116 cells were grown in McCoy's 5A Medium Modified (Gibco) supplemented with 10% fetal bovine serum (Gibco), 100 U/mL penicillin (Gibco), and 100 μg/mL streptomycin (Gibco). The antibiotics were obtained from Invitrogen (Carlsbad, CA, USA). To prepare the patient-derived single-cell suspension, tumor tissues were cut into small pieces of 1-2 mm and digested with 5 times the volume of trypsin (Sigma-Aldrich, St. Louis, MO, USA) at 37 °C for 30 min. The tissue debris was then filtered out, and cells were collected by low-speed centrifugation.

### Immunohistochemistry (IHC) staining

Tumor tissues from surgical excision were examined by standard IHC staining. Briefly, the tissues were fixed with formalin, embedded in paraffin, and then cut into 4-μm-thick sections. After deparaffinization, rehydration, and antigen retrieval, the sections were blocked with goat serum and incubated with rabbit anti-human polyclonal ALDH1 or PD-L1 antibodies (1:200 dilution; Abcam, Cambridge, UK). The sections were then counterstained with hematoxylin after incubation with the secondary antibody (Abcam). Negative control slides were treated in parallel with each batch of staining. IHC scores were calculated using Image J software according to the area and optical density of positive staining.

### Flow cytometry

To evaluate the surface expression of ALDH1, PD-L1, or anti-PD-L1 CAR, cells were washed with phosphate-buffered saline containing 2% bovine serum albumin (Sigma-Aldrich) and incubated with phycoerythrin- or allophycocyanin-labeled mouse anti-human antibodies (Cell Signaling Technology, Danvers, MA, USA). Isotype antibodies were used as control. The stained cells were evaluated using a BD FACS Vantage SE Flow Cytometer (BD Biosciences, USA), and the results were analyzed using the included software.

### ALDEFLUOR assay

An ALDEFLUOR kit (StemCell Technologies) was used to isolate ALDEFLUOR^+^/ALDH1^high^ CSCs and ALDEFLUOR^-^/ALDH1^low^ non-CSCs from patient-derived tumor cells or SW480 cells. A fluorescence substrate (BAAA), which can freely diffuse across cell membranes and be oxidized by ALDH1, was used in the ALDEFLUOR experiment. The oxidized BAAA provided a bright fluorescence signal that could be recognized by the flow cytometer. For each independent assay, diethylaminobenzaldehyde (DEAB) was used as an ALDH1 inhibitor to stain the cells as a negative control. Flow cytometry sorting gates were established by excluding propidium iodide-positive cells and ALDEFLUOR-positive cells after DEAB treatment.

### Tumorigenicity of ALDH1^high^ and ALDH1^low^ cells

Spheroid formation assays of ALDH1^high^ and ALDH1^low^ SW480 cells were conducted in ultra-low attachment 10-cm dishes. Cells (5.0 × 10^3^) were seeded, and the number of cell aggregates was calculated under a microscope after 7 days. For *in vivo* tumorigenicity tests, equal amounts of ALDH1^high^ and ALDH1^low^ SW480 cells (1.0 × 10^3^, 1.0 × 10^4^, or 1.0 × 10^5^) were mixed with Matrigel (BD Biosciences) at a 1:1 ratio and were injected into the opposite side forelegs of each mouse. Tumor size was measured after one month.

### Generation of CCSC-DC vaccine-sensitized T cells

ALDH1^high^ cells were suspended in sterile PBS at a concentration of 5 × 10^6^ cells/mL and then treated with five cycles of rapid freeze-thaw in a 37 °C water bath and liquid nitrogen to prepare the lysates. The lysates were collected by centrifugation at 2000 rpm for 7 min at 4 °C. DCs were induced from peripheral blood-derived mononuclear cells of the patients with colorectal adenocarcinoma recruited for this study. After depleting red blood cells by incubation with ammonium chloride potassium lysis buffer, the mononuclear cells were cultured at a concentration of 1 × 10^6^ cells/mL in RPMI-1640 medium supplemented with 10% heat-inactivated fetal bovine serum, 2 mM L-glutamine, 20 ng/mL granulocyte-macrophage colony-stimulating factor (GM-CSF), 20 ng/mL interleukin (IL)-4, 100 U/mL penicillin, and 100 μg/mL streptomycin. Fresh medium supplemented with GM-CSF (20 ng/mL) and IL-4 (20 ng/mL) was added on day 4. On day 6, DCs were harvested by gentle pipetting and enriched by Opti-Prep Density Gradient Medium (Stem Cell Technologies). After overnight incubation, the immature DCs were loaded with CSC lysates at a 1:1 ratio. The maturation stimulus tumor necrosis factor-α was added to the medium at 20 ng/mL after 7 days of culture to generate the CCSC-DC vaccine. Finally, T cells, which originated from identical healthy donors as DCs, were sensitized using the prepared CCSC-DC vaccine through a week of coculture at a ratio of 1:1.

### Lentivirus construction

The sequence of the anti-PD-L1 fragment was derived from the extracellular domain of PD-1. The anti-PD-L1 fragment, Fc domain, 4-1BB costimulator, and CD3ζ signaling moiety were incorporated in the pCDffH-CMV-MCS-EF1-copGFP lentivirus vector. Lentivirus plasmids were amplified using DH5α *Escherichia coli*.

### Production of PD-L1-CAR-T cells

293T cells were cotransfected with CAR plasmids and packaging plasmid (pCMV-VSVG and pCMV-DR9) using a NanoFect reagent (Alstem). The lentivirus-containing medium was collected 48 and 72 h later. To obtain a high concentration of lentivirus, a TransLv Lentivirus Precipitation Solution (Transgen) was employed. Fresh T cells from human peripheral blood were infected with the lentivirus at a multiplicity of infection of 10:1. T cells were counted every 3 days, and fresh CAR-T medium was added to maintain the cell density at 1.0-3.0 ×10^6^ cells/mL.

### *In vitro* cytotoxicity test

Two weeks after transduction, PD-L1-CAR-T cells and CCSC-DC vaccine-sensitized T cells were counted and washed with fresh medium twice. The ALDH1-positive SW480 cells were resuspended in RPMI 1640 medium (Gibco) at 1.0 × 10^6^ cells/mL. PD-L1-CAR-T cells and/or CCSC-DC vaccine-sensitized T cells (1.0 × 10^5^) were transferred into 96-well plates containing 1.0 × 10^4^ ALDH1^high^ SW480 cells (for a 10:1 effector/target ratio). After incubating for 4 h at 37 °C with 5% CO_2_, the release of lactate dehydrogenase (LDH) was measured using a kit (Roche) according to the manufacturer's instructions, and cytotoxicity was calculated.

### *In vivo* antitumor efficacy

Twenty-five (five per group) STOCK-Foxn1nu/Nju mice were subcutaneously injected with 5 × 10^6^ SW480 cells on day 0 to establish a xenograft tumor model. On days 7 and 14, the mice were intravenously treated with 3.0 × 10^6^ PD-L1-CAR-T cells, CCSC-DC vaccine-sensitized T cells, both PD-L1-CAR-T cells and CCSC-DC vaccine-sensitized T cells, normal T cells, or PBS. Tumor growth was monitored and measured using Vernier calipers every third day. One month later, surgery was conducted to remove the tumors. A caliper was used to measure the length and width of the tumor, and the tumor volume was approximated as follows: 1/2 × length × (width)^2^.

### Statistics

Statistical analyses were performed using Statistical Package for Social Science version 22.0. Results were expressed as means ± standard deviations. Two-sample t- tests were applied to compare two independent groups for continuous endpoints if normally distributed. One-way analysis of variance followed by Tukey's multiple comparisons post hoc test was used when three or more independent groups were compared. For non-normally distributed endpoints, Kruskal-Wallis test followed by a Dunn's multiple comparisons test was used. Results with P values of less than 0.05 were considered statistically significant.

## Results

### Patients' clinic parameters

A total of forty patients were included in the study. The age of all forty patients ranged from 28 to 87, and the median age was 68. There were twenty-eight males and twelve females. BMI of all the patients ranged from 16.13-29.79 with a median of 24.93. Six patients got a right colon cancer while thirty-four patients got it at left or rectum. Eleven cases were poorly differentiated and twenty-nine cases were intermediately differentiated. As for lymphatic metastasis, Thirty-three cases were N0-3 and seven were N4-6. The tumor sizes ranged from 2.2 cm - 11.0 cm with a median of 4.8 cm. The detailed clinic parameters of enrolled patients are shown in Table 1.

### PD-L1 was highly expressed in patient-derived ALDH1-positive CCSCs

**Figure 1:**
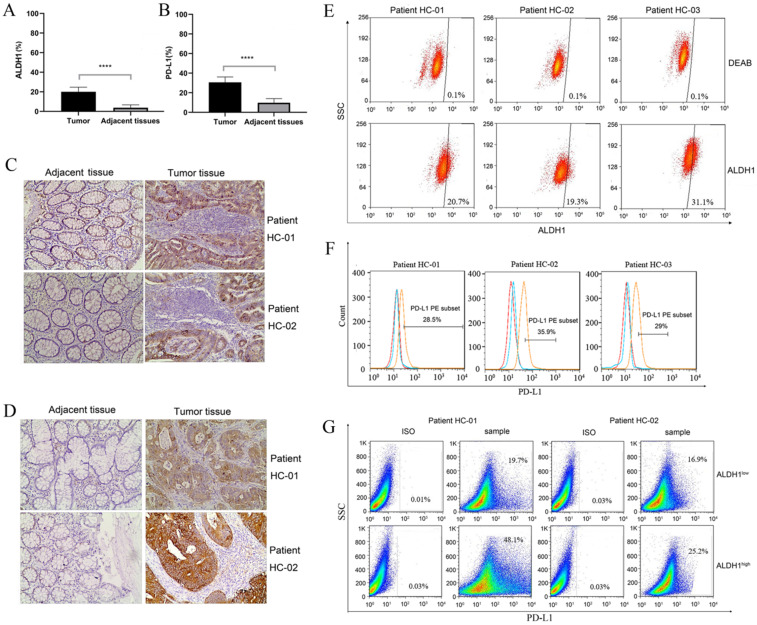
** ALDH1 and PD-L1 expression in tumors and adjacent tissues of patients with colorectal cancer. (A)** Statistical analysis of ALDH1 expression in colorectal tumor tissues and adjacent tissues. N = 40 in each group. **(B)** Statistical analysis of PD-L1 expression in colorectal tumor tissues and adjacent tissues. N = 40 in each group. **(C)** Representative images of IHC staining for ALDH1 in samples of noncancerous tissues as well as cancerous lesions. **(D)** Representative images of IHC staining for PD-L1 in samples of noncancerous tissues and cancerous lesions. **(E)** ALDH1 expression in dissociated carcinoma cells from patients HC-01, HC-02, and HC-03, as determined by flow cytometry. **(F)** PD-L1 expression in dissociated carcinoma cells from patients HC-01, HC-02, and HC-03, as determined by flow cytometry. **(G)** Dissociated cancer cells were sorted into ALDH1^high^ and ALDH1^low^ cells using an ALDEFLUOR kit, and PD-L1 expression was evaluated in ALDH1^high^ and ALDH1^low^ cells from patients HC-01 and HC-02. IHC: immunohistochemistry. ^****^P < 0.001.

ALDH1 is a biomarker of CCSCs. Therefore, we first evaluated the expression of ALDH1 in clinical samples by IHC. As shown in Figure 1A and Figure 1C, colorectal tumor tissues showed significantly higher ALDH1 expression than the normal colonic epithelium (P < 0.0001). This result confirmed the presence of CCSCs in colorectal tumor tissues. Subsequently, we evaluated PD-L1 expression. Both the positive area and intensity of PD-L1 in tumor sections were markedly higher than those in epithelial tissue sections (P < 0.0001, Figure 1B, D). In tumor tissues, the expression score of PD-L1 reached 27.23 ± 3.45, whereas that in the control group was only 7.48 ± 3.80.

In order to further verify the relationship between ALDH1 and PD-L1, tumor samples from three typical ALDH1^high^/PD-L1^high^ patients were dissociated into single cells. Flow cytometry results showed that all three patients had high proportions of ALDH1^+^ subsets and PD-L1^+^ subsets (Figure 1E, F). The ratios of ALDH1^+^ cells in patients HC-01, HC-02, and HC-03 were 20.7%, 19.3%, and 31.1%, respectively, compared with those in DEAB-treated cells. As an immunosuppressive molecule, PD-L1 is often highly expressed in tumor cells. The expression of PD-L1 of patients HC-01, HC-02, and HC-03 reached 28.5%, 35.9%, and 29.0%, respectively. In addition, we sorted the dissociated tumor cells into ALDH1^high^ and ALDH1^low^ subpopulations. There were more PD-L1-positive phenotype cells in ALDH1^high^ subsets than in ALDH1^low^ subsets (Figure 1G). Up to 48.1% and 25.2% of PD-L1-positive cells were detected in ALDH1^high^ subsets from patients HC-01 and HC-02, respectively, whereas only 19.7% and 16.9% of the cells, respectively, were detected in the ALDH1^low^ subsets. This result indicating that the expression of PD-L1 in CCSCs was correlated with that of ALDH1 and that ALDH1^high^ CCSCs may exhibit high PD-L1 expression.

### PD-L1 was highly expressed in cell line-derived ALDH1-positive CCSCs

**Figure 2:**
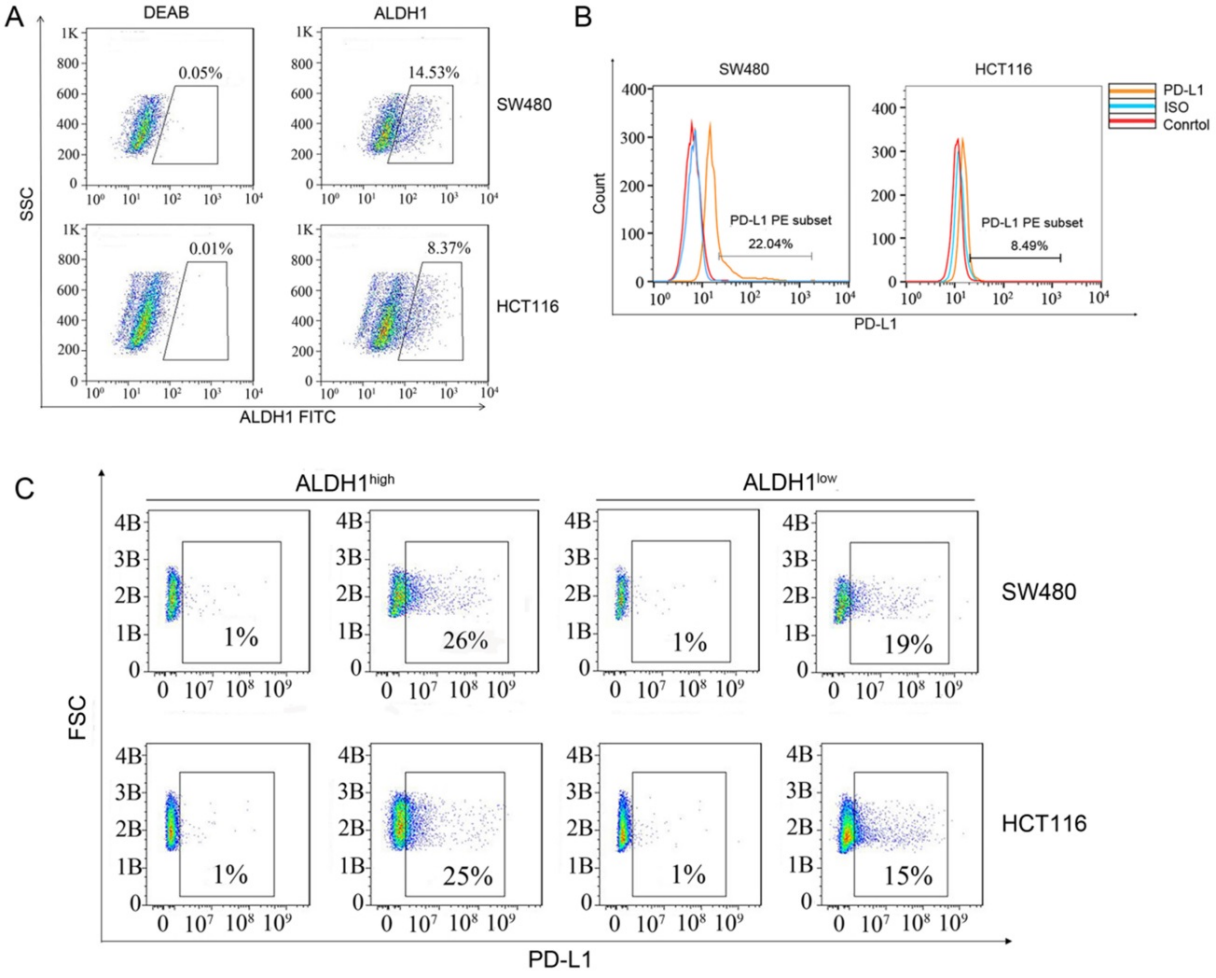
** ALDH1 and PD-L1 expression in SW480 and HCT116 cells. (A)** ALDH1 expression in SW480 and HCT116 cells.** (B)** PD-L1 expression in SW480 and HCT116 cells. **(C)** SW480 and HCT116 cells were divided into ALDH1^high^ and ALDH1^low^ cells using an ALDEFLUOR kit, and the expression of PD-L1 was evaluated in ALDH1^high^ and ALDH1^low^ cells. B: Billion.

Next, we used flow cytometry to check the proportions of ALDH1-positive CCSCs in SW480 and HCT116 CRC cells. Approximately 14.53% of SW480 cells and 8.37% of HCT116 cells were found to express ALDH1, indicating that CCSCs were relatively abundant (Figure 2A). However, to our surprise, the proportion of PD-L1-positive cells among SW480 cells was 22.04% (Figure 2B), which was much higher than that in HCT116 cells (8.49%). We sorted out the ALDH1^high^ CCSCs from SW480 and HCT116 cells and measured their PD-L1 expression. Approximately 26% of ALDH1^high^ SW480 cells and 25% of ALDH1^high^ HCT116 cells showed the PD-L1-positive phenotype, whereas only 19% of ALDH1^low^ SW480 cells and 15% of ALDH1^low^ HCT116 cells showed this phenotype (Figure 2C). These results confirmed the high expression of PD-L1 in CCSCs.

### Generation of PD-L1-CAR-T cells

**Figure 3:**
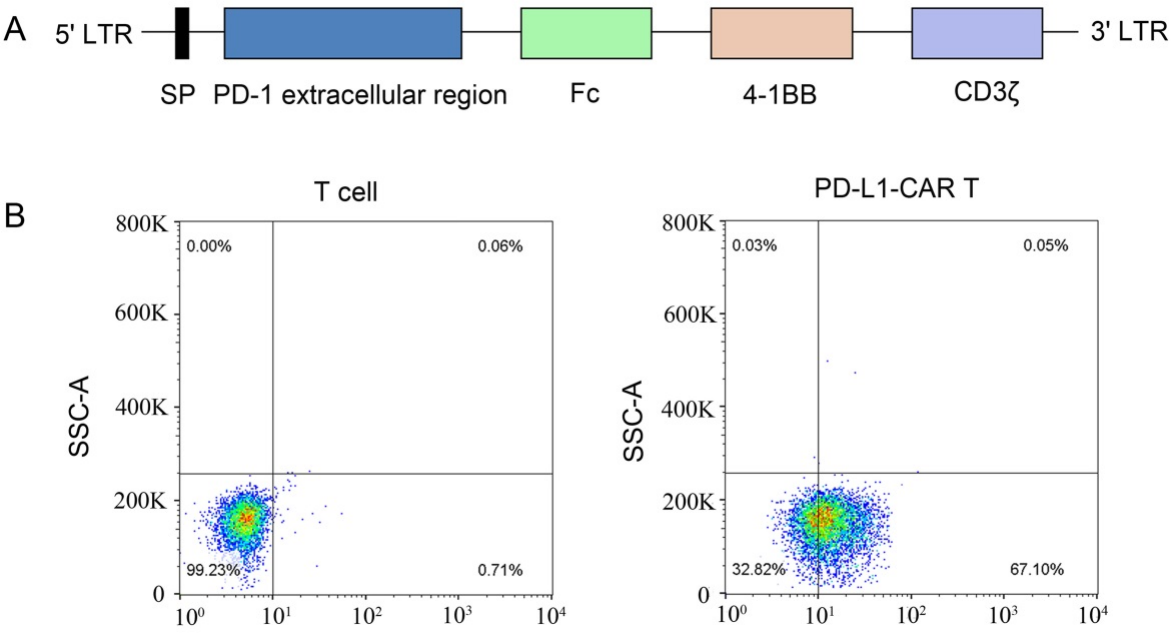
** Construction of the PD-L1-CAR molecule and preparation of CAR-T cells. (A)** Schematic diagram of the second-generation PD-L1 CAR lentivirus construct containing a PD-1 extracellular region, an Fc domain, a 4-1BB costimulator, and a CD3ζ region. **(B)** T cells from human peripheral blood were activated with CD3/CD28 beads and transfected with the CAR-containing lentivirus. CAR expression was detected using phycoerythrin (PE)-labeled anti-human PD-1 antibodies. Nontransfected T cells were used as the control.

A second-generation PD-L1-specific CAR constructs containing a PD-1 extracellular region, an Fc domain, a 4-1BB costimulator, and a CD3ζ signaling moiety was generated and inserted into a lentiviral vector (Figure 3A). Anti-CD3/CD28 monoclonal antibody bead-activated primary T cells from a healthy donor were transduced with lentiviral particles encoding the PD-L1-CAR vector or empty vector. The expression of PD-L1-CAR-T cells was determined by staining the cells with a mouse anti-human antibody recognizing PD-1. Flow cytometry showed that approximately 67.1% of transduced cells expressed CAR; that in empty vector-transduced cells was undetectable (Figure 3B).

### Synergistic killing effects of CCSC-DC-sensitized T cells and PD-L1-CAR-T cells *in vitro*

**Figure 4:**
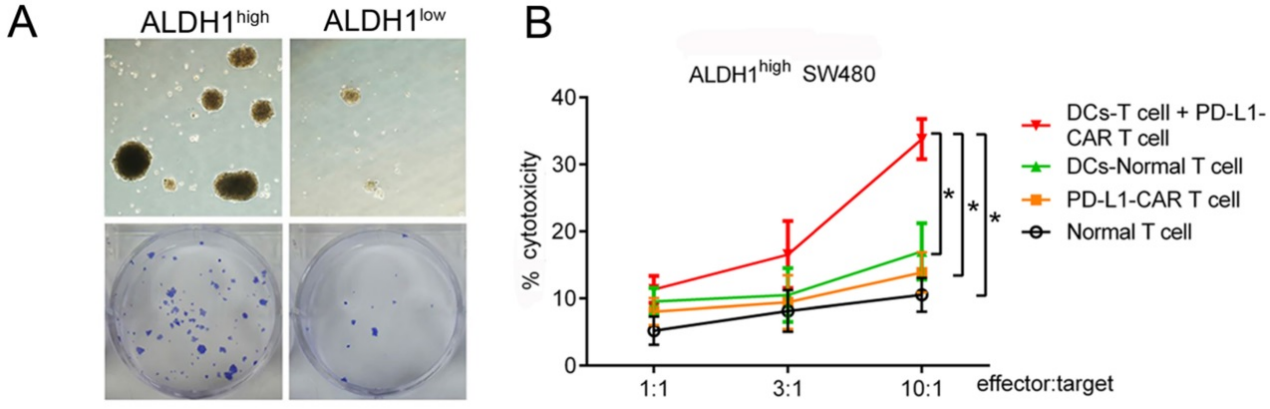
** PD-L1-CAR-T cells combined with CCSC-DC vaccine-sensitized T cells markedly inhibited the growth of ALDH1^high^ SW480 cells. (A)** ALDH1^high^ SW480 cells grew rapidly in dishes during a 7-day culture. **(B)** Cytotoxicities of different treatments at different E/T ratios. After incubating for 4 h, cytotoxicity was measured by the LDH release method. Each assay was repeated independently five times. E/T: effector:target, LDH: lactate dehydrogenase. ^*^P < 0.05.

Based on our finding that PD-L1 was highly expressed in ALDH1-positive CCSCs, we designed a combinational therapy strategy that used CCSC-DC vaccine-sensitized T cells and PD-L1-CAR-T cells to kill both CCSCs and normal tumor cells. To produce the vaccine, CCSCs were lysed by rapid freezing and thawing and then incubated with human peripheral blood-derived DCs. Human T cells were then sensitized using the vaccine. The cytotoxicity of the combinational therapy was tested using ALDH1^high^ CCSCs isolated from SW480 cells. First, we evaluated the proliferation rates of the isolated ALDH1^high^ CCSCs and ALDH1^low^ normal cells. The ALDH1^high^ CCSCs showed strong reproduction ability, with a 5.76-fold increase in number compared with ALDH1^low^ CCSCs after culturing for 7 days (Figure 4A).

At the E/T ratio of 10:1, the PD-L1-CAR-T cells exhibited modest cytotoxicity to ALDH1^high^ CCSCs. However, there were no significant differences compared with T cells (13.88% versus 10.56%, P > 0.05). On the other hand, CCSC-DC vaccine-sensitized T cells showed higher cytotoxicity against ALDH1^high^ cells at the same ratio (17.00%, P < 0.05, Figure 4B). Notably, the combinational therapy showed significantly more powerful cytotoxicity against the CCSCs than the two monotherapies. The killing efficiency of the combined treatment was 243.4% (33.79% versus 13.88%) higher than that of the PD-L1-CAR-T cell group (Figure 4B).

### Synergistic killing effects of CCSC-DC-sensitized T cells and PD-L1-CAR-T cells *in vivo*

**Figure 5:**
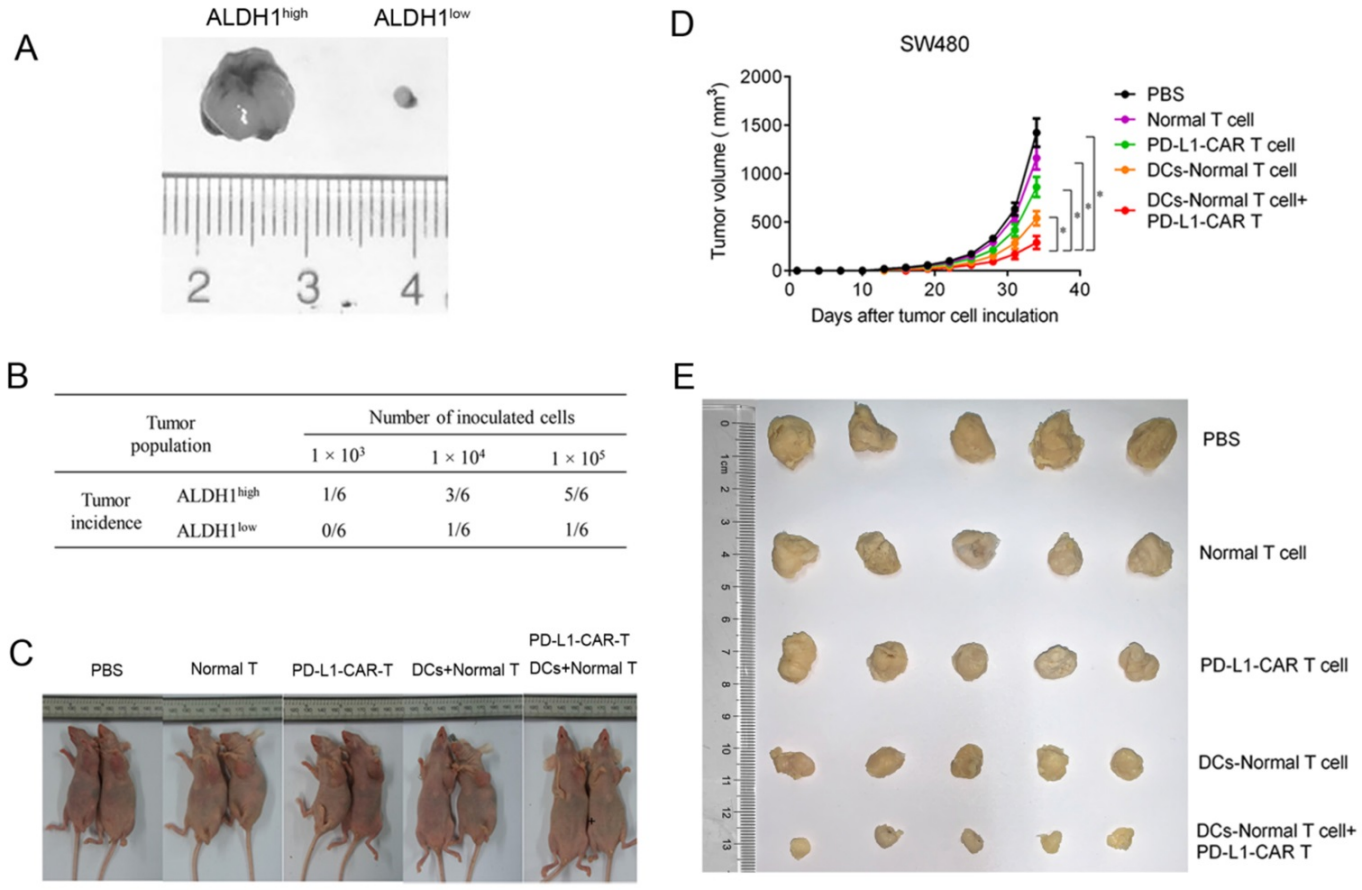
** PD-L1-CAR-T cells combined CCSC-DC vaccine-sensitized T cells inhibited colorectal tumor growth *in vivo*. (A)** Sizes of ALDH1^high^ SW480 tumors and ALDH1^low^ tumors. **(B)** Tumorigenicity test using ALDH1^high^ SW480 cells and ALDH1^low^ SW480 cells. Equal amounts of cells (1.0 × 10^3^, 1.0 × 10^4^, or 1.0 × 10^5^) were injected into the opposite side forelegs of mice. The tumor incidence was evaluated one month later. **(C)** Tumor growth after mice received normal T cells, CAR-T cells, vaccine-sensitized T cells, CAR-T cells combined with vaccine-sensitized T cells, or PBS. **(D)** Tumor volumes were measured every third day from day 0 after intravenous injection with normal T cells, CAR-T cells, vaccine-sensitized T cells, CAR-T cells combined with vaccine-sensitized T cells, or PBS until day 33. Five mice were included in each group. **(E)** Representative images of tumors after mice received normal T cells, CAR-T cells, vaccine-sensitized T cells, CAR-T cells combined with vaccine-sensitized T cells, or PBS. ^*^P < 0.05.

To examine the antitumor potential of combining CCSC-DC vaccine-sensitized T cells and PD-L1-CAR-T cells, an *in vivo* xenograft tumor model was established using SW480 cells. The growth rate of ALDH1^low^ tumors was much lower than that of ALDH1^high^ tumors (both with 1.0 × 10^5^ cells) after a 1-month observation period (Figure 5A). In addition, the tumor incidence of ALDH1^low^ cells was extremely low (Figure 5B). Only one of six mice formed tumor masses, even after inoculation of a relatively high number of ALDH1^low^ cells (1.0 × 10^5^). However, five of six mice in the ALDH1^high^ CCSC group exhibited tumorigenesis.

We then evaluated the antitumor efficacy of the combination therapy in the xenograft model (Figure 5C). Tumor remission in mice receiving normal T-cell injections was negligible 33 days after treatment compared with mice administered PBS. PD-L1-CAR-T monotherapy moderately relieved the tumor burden. However, the effects were inferior to monotherapy with CCSC-DC vaccine-sensitized T cells. Notably, combination therapy dramatically decreased the tumor burden and volume in mice compared with the PBS control group (Figure 5D, 5E).

## Discussion

In 2007, O'Brien and Ricci-Vitiani discovered CCSCs in two independent studies 20,21. Researchers have shown that conventional chemotherapeutic drugs and radiotherapy cannot completely clear CCSCs owing to various known and unknown intrinsic mechanisms, some of which include abnormal activation of proliferation-related signaling pathways, such as Wnt, Notch, and Hedgehog; dormancy induced by treatment intervention; protection of the tumor microenvironment; and conversion of non-CCSCs to CCSCs 22. Thus, CCSCs are still a significant concern for patients owing to poor therapeutic responses and prognoses.

In this study, we found that ALDH1 was highly expressed in the tumor tissues of patients with CRC. We previously demonstrated that administration of ALDH^high^ SCC7 CSC-DC vaccines in immunocompetent mice induces protection against subsequent SCC7 challenge 23. The ALDH^high^-DC (CSC-DC) vaccine significantly reduced tumor recurrence compared with the PBS control, normal-DC, and ALDH^low^-DC vaccination, respectively. In a metastatic D5 mouse melanoma model, we proved that T cells harvested from ALDH^high^ DC-vaccinated animals selectively and significantly killed the ALDH^high^ D5 cells compared with the ALDH^low^-DC group. By contrast, CTLs generated from splenocytes of mice subjected to ALDH^low^ DC and normal-DC vaccination selectively killed ALDH^low^ D5 cells with minimal cytotoxic effect on ALDH^high^ cells. The above results suggested that DC pulsed with lysate antigens from CSCs or non-CSCs would summon different lethal effects. In the treatment of colorectal cancer, CSCs were also endowed with self-renewing and tumor-initiating properties and responsible for resistance to chemotherapy. Based on our previous study and the urgency of treating CSCs, we mainly evaluated the anti-CSCs (the ALDH^high^ subgroup of colorectal cancer cells) potential of vaccination with PDL-1 CAR-T therapy. Indeed, the expression of ALDH1 in tumor tissues was four times higher than that in normal tissues, suggesting the potential abundance of CCSCs in tumor masses. High levels of CSCs are associated with rapid tumor growth and progression. In an *in vitro* model, Escobar et al. showed that CSCs had a higher expansion rate than non-CSCs, even under a low cell seeding density 24. Similarly, in our study, ALDH1^high^ CCSCs showed increased reproduction ability compared with ALDH1^low^ CCSCs after culturing for 7 days. Moreover, we tested the tumorigenicity of ALDH1^high^ cells *in vivo* and showed that ALDH1^high^ cells had an extremely high tumor incidence and grew much faster than ALDH1^low^ cells. It is worth noting that although ALDH1 was one of prominent biomarkers in CCSCs, staining of ALDH1 alone might not equal to the total number of CCSCs. In other words, the lack of stem cell validation with CD133 and CD116 as a potential limitation to the present study.

PD-L1 binding to its receptor PD-1 is critical for tumor cells to escape from immune surveillance via inhibition of T cell function. Additionally, PD-L1 has been shown to promote CCSC amplification by activating multiple signaling pathways 25, 26. High PD-L1 levels are always associated with poor clinical outcomes 27. In this study, we isolated ALDH1-positive CCSCs from tumor tissues of patients and checked PD-L1 expression in these cells; our results showed that the positive rate of PD-L1 was up to 48.1%, whereas that of non-CCSCs was less than 20%, indicating that PD-L1 expression was high in ALDH1-positive CCSCs. Furthermore, we found that the expression of PD-L1 in ALDH1-positive CCSCs was also 7-10% higher than that of non-CCSCs.

CAR-T therapy has shown great success in treating hematologic malignancies, and the number of clinical trials of CAR-T therapy has increased dramatically worldwide 28. Advances are also being made in the treatment of solid tumors. The main targets of CAR-T therapy for solid tumors include ganglioside GD2 in neuroblastoma, mesothelin in pancreatic cancer, carcinoembryonic antigen in CRC, fibroblast activation protein in malignant pleural mesothelioma, and epidermal growth factor receptor in non-small cell lung cancer 29. Since we detected high expression of PD-L1 in ALDH1-positive CCSCs, we designed an approach for PD-L1-CAR-T cell therapy. Unfortunately, PD-L1-CAR-T cells showed modest antitumor efficacy; the lack of potent efficacy may be attributed to the effects of the tumor microenvironment and barrier of solid tumors on monotherapies.

DCs are the most potent antigen-presenting cells, making them good candidate vaccines for CSCs. Available evidence suggests that DC vaccines can induce significant antitumor responses by inducing host immunity 30, 31. This type of treatment has been shown to be a promising immunotherapy for cancers. For instance, the first US Food and Drug Administration-approved cancer vaccine Sipuleucel-T was developed from autologous DCs loaded with the engineered fusion protein of prostatic acid phosphatase and GM-CSF; this vaccine showed encouraging results in prolonging median survival in patients with castration-resistant prostate cancer 32. In this study, we immunized DCs with CCSC lysates and produced a CCSC-DC vaccine. T cells sensitized to the CCSC-DC vaccine showed moderate cytotoxicity in colorectal tumor cells, resulting in a killing efficiency of 17.0%. However, these results were not as exciting as expected. Compared with Sipuleucel-T, we used lysates of CCSCs instead of single fusion protein molecules to prepare the DC vaccine; this may have reduced the specificity and efficacy of the vaccine.

Single immunotherapies, including both CAR-T cells and tumor vaccines, have shown inadequate efficacy in treating malignant tumors 33. Notably, DC-CIK (dendritic cells-cytokine induced killer) therapy has been proved to be less effective in the clinical treatment of cancers, which is indeed the conclusion of most experiments. However, the first clinical study using the CIK method was conducted in 1999 and included 7 patients with CRC, 2 with lymphomas and 1 with renal cancer. One lymphoma-patient showed complete remission, six of the other had a progress, three no changes 34. A recent phase 2 trial applied CIK-treatment to CRC (colorectal cancer) patients combined with FOLFOX vs. FOLFOX alone. The 3-year OS was significantly improved from 23% in the control group to 48% in the CIK-FOLFOX group. There was no significant difference in side effects between the groups 35. This showed that the adjuvant treatment of DC-CIK therapy has a good effect on the prognosis of CRC. Here, we examined the antitumor potential of coapplication of CCSC-DC vaccine-sensitized T cells and PD-L1-CAR-T cells, and found that combination therapy markedly killed cancer cells and relieved the tumor burden in mice. Nevertheless, further research is needed, such as a vaccination with unrelated tumor cells would have been useful as a control.

In addition, the lack of relevant IHC analysis of the mouse tumor after the tumor was removed is a potential limitation to the present study. Although our animal experiments were successful, there are still many uncertainties for potential clinical application. For example, we used immunodeficient mice in this study; however, immunodeficiency does not always mean that animals do not have an immune response to foreign molecules. The tumor cells of human origin and the human PD-1 molecule used in this study may produce a host-versus-graft response, and the T cells (human origin) may produce both a host-versus-graft response and graft-versus-host responses. For the CAR structure design, we used the extracellular region of PD-1 to recognize the PD-L1 molecule, rather than a monoclonal antibody against PD-L1. Sufficient evidence indicating which strategy is better is not available, and further analyses of the affinity for PD-L1, the tumor-killing effect, immunogenicity, *in vivo* amplification, and formation of central memory cells are required. Accordingly, these factors should be optimized in future studies. Additionally, we plan to use humanized animals and tumor cells to reduce the immune response caused by foreign components in order to improve the reliability of the experimental results. The molecular structure of CAR should also be optimized in order to obtain CAR-T cells with high cytotoxicity and long survival times. Moreover, therapies targeting tumor stem cells and PD-1/PD-L1 blocking therapies are currently both very promising directions in tumor immunotherapy. Hence, it will be necessary to evaluate the combined application of tumor stem cell vaccines and PD-1/PD-L1 blocking antibodies in the clinical setting.

In summary, we demonstrated that the expression of PD-L1 in ALDH1-positive CCSC subpopulations was higher than that in non-CCSC subpopulations. Moreover, the combination therapy that we developed using PD-L1-CAR-T cells and CCSC-DC vaccine-sensitized T cells had the potential to suppress CRC. Thus, our findings may contribute to the clinical development of new strategies for CRC treatment.

## Figures and Tables

**Table 1:**
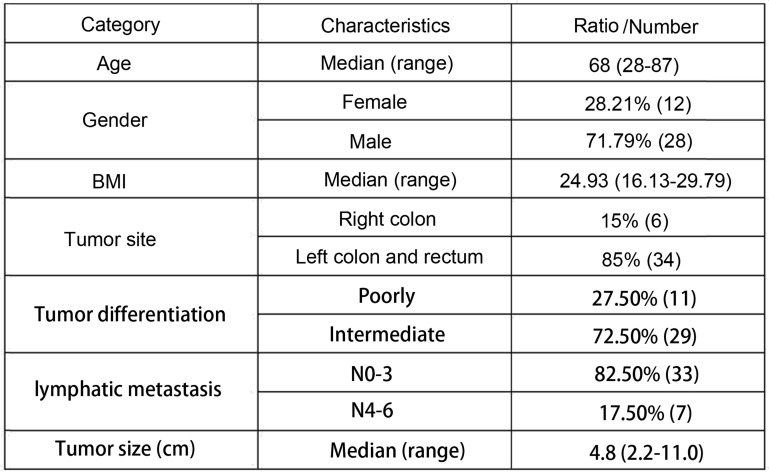
Basic clinic parameters of all 40 patients

BMI: Body Mass Index.
